# Network analysis through the use of joint-distribution entropy on EEG recordings of MCI patients during a visual short-term memory binding task

**DOI:** 10.1049/htl.2018.5060

**Published:** 2019-03-29

**Authors:** Alexandra Josefsson, Agustín Ibáñez, Mario Parra, Javier Escudero

**Affiliations:** 1 School of Engineering, Institute for Digital Communications, The University of Edinburgh, EH9 3FB, Edinburgh, UK; 2 Institute of Cognitive and Translational Neuroscience (INCYT), INECO Foundation, Favaloro University, Buenos Aires, Argentina; 3 National Scientific and Technical Research Council (CONICET), Buenos Aires, Argentina; 4 Center for Social and Cognitive Neuroscience (CSCN), School of Psychology, Universidad Adolfo Ibanez, Santiago, Chile; 5 Universidad Autónoma del Caribe, Barranquilla, Colombia; 6 Centre of Excellence in Cognition and its Disorders, Australian Research Council (ACR), Sydney, Australia; 7 School of Psychological Sciences and Health, University of Strathclyde, Glasgow, UK

**Keywords:** electroencephalography, medical signal processing, diseases, neurophysiology, brain, entropy, cognition, Alzheimer's disease, mild cognitive impairment, electroencephalogram recordings, brain activity, high temporal resolution, brain functional connectivity, functional network, beta-filtered EEG recordings, short-term memory binding task, MCI patients, EEG functional connectivity changes, network differences, network analysis, joint-distribution entropy, early diagnosis

## Abstract

The early diagnosis of Alzheimer's disease (AD) is particularly challenging. Mild cognitive impairment (MCI) has been linked to AD and electroencephalogram (EEG) recordings are able to measure brain activity directly with high temporal resolution. In this context, with appropriate processing, the EEG recordings can be used to construct a graph representative of brain functional connectivity. This work studies a functional network created from a non-linear measure of coupling of beta-filtered EEG recordings during a short-term memory binding task. It shows that the values of the small-world characteristic and eccentricity are, respectively, lower and higher in MCI patients than in controls. The results show how MCI leads to EEG functional connectivity changes. They expect that the network differences between MCIs and control subjects could be used to gain insight into the early stages of AD.

## 1 Introduction

It is useful to identify people with mild cognitive impairment (MCI) as they would be at higher risks of developing Alzheimer's disease (AD). This is important given that, nowadays, 35.6 million people live with dementia worldwide [1], posing a big strain on the economy and society. Furthermore, as the world has an ageing population, the number of people affected by dementia is expected to increase significantly in the future [1].

Further understanding of MCI could contribute to developing an earlier diagnosis of AD, something that could help dementia patients and their caregivers to make better, informed decisions about their lives. An earlier diagnosis means earlier access to information and support [2]. There are medical benefits of an early diagnosis of dementia too. The patient can obtain earlier access to therapies to improve their quality of life. The earlier diagnosis also means that patients can take part in further research [2].

The analysis of brain activity via the processing of electroencephalogram (EEG) recordings is a promising avenue to characterise MCI and early AD. In an EEG, electrodes are placed on the surface of the scalp to record the electrical activity generated by groups of neurons in the brain. When a neuron is activated, an electrical signal is transmitted between nerve cells at the synapse. From here, the signal is conducted to the cell body, along the axon and finally to the axon terminal where the neuron synapses with a new cell. For this conduction to happen, ion channels transport ions through the cell membrane, both at the axon and at the synapse [3]. As the electrodes are placed on the patient's scalp, larger groups of active neurons will produce EEG signals that can be seen in recordings [3]. EEG signals were chosen as the way to measure electrical signals, due to being a non-invasive method, and being a portable method to use. EEG recordings also have very high temporal resolution which is desirable for detecting rapid changes in brain activity [4].

Some frequency bands of the EEG signals are of particular interest in different applications. Among them, we focus on the alpha band at 8–13 Hz and the beta band at 13–30 Hz. Alpha activity has been prominent in the study of AD and higher-frequency bands (e.g. beta) have been associated with cognitive processes.

Brain graphs (or networks) are mathematical representations of (structural or functional) interactions in the brain [5]. Such networks can be produced from EEG recordings. Here each electrode can be represented as a node in the network. The edges between the nodes are defined by coupling between the EEG signals. Networks are a way to represent complex systems, which the brain's structural and functional systems can be considered [6]. The production of brain graphs enables the analysis of EEG signals due to the generalisability and interpretability of brain graphs [5]. When analysing brain graphs, network parameters are compared.

Indeed, there have been several findings of how network parameters in brain graphs have been linked to various diseases. The ones of most interest to this Letter are ones concerning MCI and dementia, of which the latter many studies have been done with focus on AD.

There are numerous network parameters. Some of the most commonly used are clustering coefficient, characteristic path length, small-world phenomenon, and eccentricity. The clustering coefficient indicates how much the nodes of the network tend to create tightly related groups. It measures the proportion of neighbours of a node that is also direct neighbours of each other. The characteristic path length is the average shortest path length of the network. Thus, well-integrated networks have low characteristic path lengths. Networks are deemed to exhibit small-world phenomenon when they are seen to have a high clustering coefficient and a comparatively smaller characteristic path length, when compared with a random network [7]. Thus, the small-world characteristic is a measure of the balance between segregation and integration in a network. Eccentricity is the maximum shortest path length between any two nodes in the network [8].

In previous studies, brain networks of AD patients, compared with controls, have been reported to have longer characteristic path lengths in the beta band and lower small-world characteristics [9]; and lower small-world characteristics in networks based on cortical thickness [10]. Eccentricity in the alpha band was reported to be higher amongst AD patients than subjective cognitive decline patients [11], which has been hypothesised as an earlier indication of AD than MCI [12]. In a study comparing AD and patients with frontotemporal lobar degeneration (FTLD) and control subjects, a lower clustering coefficient was seen in the lower alpha and beta band of AD compared with other subjects. The characteristic path length was shorter in the alpha band of AD patients. The AD patients were seen to have lower small-world characteristics than the control and FTLD subjects [13]. In a study looking at magnetoencephalogram recordings of AD patients during a no-task, eyes-closed condition, it was found that AD patients had a lower clustering coefficient in the lower alpha band and path length than control patients [14]. It has, however, been seen that eccentricity has become higher in the frontal and temporal regions for Parkinson's disease patients as the disease progressed when looking at the alpha band [15]. As Parkinson's disease patients are more likely to develop dementia [16], the results of this Letter may be of interest.

The main objective of this Letter was to find whether any differences in the beta band could be seen between the MCI and control subjects during a relevant short-term memory binding task. The dataset used has been previously described [17]. These differences were then evaluated from a network point of view. The brain networks are produced by applying a nonlinear coupling algorithm to the signals.

## 2 EEG recordings

The data analysed in this Letter comes from EEG recordings of patients subjected to a test. The subjects consisted of 13 patients with MCI. There were also 19 control subjects who did not have MCI. Five of these subjects’ data were removed from analysis as the recordings were deemed noisy when used for other purposes. This leaves a control group of 14 control subjects.

The EEG recordings used in this Letter are of when these patients were asked to recall an image they had been shown previously in a visual short-term memory binding tasks [17]. Of the experiment, we consider the binding condition. For additional details, the reader is referred to [17]. The recordings were achieved using an EEG montage with 128 channels.

The recordings were sampled at a frequency of 256 Hz over 1.40 s, with 0.2 s pre-stimuli. This pre-stimulus section was removed to focus only on the activity elicited by the task, which leaves 1.20 s of data. The EEG recordings were epoched with the number of epochs ranging from 23 to 87 for each subject.

The recordings were preprocessed using standard toolboxes [18] in order to remove artefacts and noisy epochs. The same dataset has been used before and is further described there [17].

Each sample in a channel was averaged across the epochs for the subject. After this, the beta band of the EEG recordings was extracted through filtering. The recordings were cascaded through a high-pass and low-pass Butterworth infinite impulse response (IIR) filter. Butterworth IIR filters were used due to their maximally flat passbands in order to minimise distortions to the signal as distortions in the passband could affect the nonlinear coupling between the signals. In addition, a zero-phase delay was achieved for each filter by two-pass filtering [19]. The signal is first two-pass filtered through a Butterworth IIR high-pass filter of filter order 4 and a half power frequency of 9 Hz. The output from the high-pass filter is then two-pass filtered through a Butterworth IIR low-pass filter of filter order 8 and half power frequency of 34 Hz.

## 3 Methods

The joint distribution entropy method [20] was used to find the coupling between each two channels in order to produce an adjacency matrix representing the network. Thus, the adjacency matrix produced is a square matrix corresponding to the coupling between 128 channels.

Nonlinear methods can detect coupling in physiological systems, but many require long time series, which the EEG recordings used here are not, particularly when recorded during tasks such as the visual short-term memory binding. However, the joint distribution entropy method has shown promising results in detecting weak coupling in short physiological series [20]. Therefore, this method was used. The method is briefly described below. For additional details, the reader is referred to [20].

The filtered data in each channel are first rescaled according to

(1) $$\bar u_\varphi \lpar i\rpar = \displaystyle{{u_\varphi \lpar i\rpar - \min \lpar u_\varphi \rpar } \over {\max ({u_\varphi } )- \min ({u_\varphi } )}}\eqno\lpar 1\rpar $$

where $\bar u_\varphi \lpar i\rpar $ denotes the rescaled signal data of a temporal sample *i* and $\varphi $ denotes the channel that is being looked at. The filtered data of a temporal sample in a channel before being rescaled is denoted as $u_\varphi \lpar i\rpar $ and $\min ({u_\varphi } )$ and $\max \lpar u_\varphi \rpar $ are the minimum and maximum values within the data channel $u_\varphi $ across all temporal samples. This rescaling leads to the data in each channel being in the range 0–1.

The state space can then be constructed. The state space ${\bi X}_{\bi \varphi} {\bi \lpar i\rpar} $ is given by

(2) $${\bi X}_{\bi \varphi} {\bi \lpar i\rpar} = \left[{{\bar u}_\varphi \lpar i\rpar \comma \; \, {\bar u}_\varphi ({i + \tau _\varphi } )\comma \; \, \ldots \comma \; \, {\bar u}_\varphi ({i + ({m_\varphi - 1} )\tau _\varphi } )} \right]\eqno\lpar 2\rpar $$

where $m_\varphi $ is the embedding dimension and $\tau _\varphi $ is the time delay of the channel. Each channel's rescaled samples are delayed. The delay is needed to reconstruct the attractor of the signal recorded at that channel following Takens theorem [21]. The delay ranges from 0 to $\lsqb \lpar m_\varphi - 1\rpar \tau _\varphi \rsqb $ across the samples. The bivariate state-space reconstruction considers the fact that there may be a time delay between related signals, as it takes time for different parts of the brain to communicate with each other. In this case, $\tau _\varphi $ was considered to be 1 sample, and $m_\varphi $ has been set to 2. These parameters were chosen since they have been seen to work in nonlinear analysis of EEG signals in AD with the similar methods SampEn and ApEn [22].

$\bi X_\varphi \lpar i\rpar $ is performed for samples $\lpar i = 1\comma \; \, 2\comma \; \, 3\comma \; \, \ldots \comma \; \, N - n\rpar $, where *N* is the total number of samples. The constant *n* is determined in the following manner: $n = \max ({m_\varphi } )\max ({\tau _\varphi } )$.

### 3.1 Joint distance matrix construction

First, distance matrices are found for each of the channels. A distance matrix $\bar{\bi D}_\varphi $ for channel $\varphi $ is defined by

(3) $$\bar{\bi D}_\varphi = \left\{{{\rm \Vert }{\bi X}_{\bi \varphi} {\bi \lpar i\rpar} \comma \; \, {\bi X}_{\bi \varphi} {\bi \lpar j\rpar} {\rm \Vert \bond }\, \, {\rm for}\, \, i\comma \; \, j = 1\comma \; \, 2\comma \; \, \ldots \comma \; \, \lpar N - n\rpar } \right\}\eqno\lpar 3\rpar $$

where $\Vert \cdot \Vert $ denotes the maximum norm. $\overline {\bi D} _\varphi $ gives the maximum distance between all samples within the specified channel.

The maximum distance is found between two columns of the state-space matrix assuming that the columns of the matrix contain the rescaled value at a sample *i* to $i + \lpar m_\varphi - 1\rpar \tau _\varphi $, i.e. the column contains the rescaled value at that sample as well as the corresponding delays. The maximum distance is then found between two such columns of the state-space matrix. This is done by

$$\eqalign{\Vert\bi X_\varphi \lpar i\rpar \comma \; \, X_\varphi \lpar j\rpar \Vert & =\max [{\max {\bi \lpar X_\varphi \lpar i\rpar \rpar} - \min {\bi \lpar X_\varphi \lpar j\rpar \rpar} \comma \;} \cr & \quad {\max {\bi \lpar X_\varphi \lpar j\rpar \rpar} - \min {\bi \lpar X_\varphi \lpar i\rpar \rpar }} ]} $$

for a single sample *i* and sample *j*. This distance matrix is produced for each channel such that a joint distance matrix can be produced between two channels.

A symmetric distance matrix, called the joint distance matrix $\overline {{\bi JD}} $, for two channels is given by

(4) $$\overline {{\bi JD}} = \bar{\bi J} - \sqrt {\left({\bar{\bi J} - {\bar{\bi D}}_1} \right)\left({\bar{\bi J} - {\bar{\bi D}}_2} \right)} \eqno\lpar 4\rpar $$

where $\bar{\bi D}_1$ and $\bar{\bi D}_2$ are distance matrices of two channels and $\bar{\bi J}$ is an all-ones matrix of same size as $\bar{\bi D}_\varphi $. $\overline {{\bi JD}} $ is created for all pairs of channels. This represents the joint distance between the pairs of channels.

### 3.2 Probability density estimation

The next step is to produce a probability density estimation from the distance of all elements, except the diagonal in the joint distance matrix. The diagonal is excluded from this calculation as this represents connections the sample has with itself.

First, the number of bins is calculated using Doane's formula

(5) $$B = 1 + \mathop {\log }\nolimits_2 \, n_{{\rm obv}} + \mathop {\log }\nolimits_2 \left({ 1 + \displaystyle{{\vert g_1\vert } \over {\sigma _{g1}}}} \right)\eqno\lpar 5\rpar $$

where *B* is the number of histogram bins; $g_1$ is the skewness of $\overline {{\bi JD}} $; and $n_{{\rm obv}}$ is the number of observations. The number of observations $n_{{\rm obv}}$ is given by

(6) $$n_{{\rm obv}} = \left({N - n} \right)^2 - \left({N - n} \right).\eqno\lpar 6\rpar $$

$\sigma _{g1}$ is defined as

(7) $$\sigma _{g1} = \sqrt {\displaystyle{{6\left({n_{{\rm obv}} - 2} \right)} \over {\left({n_{{\rm obv}} + 1} \right)\left({n_{{\rm obv}} + 3} \right)}}} .\eqno\lpar 7\rpar $$

The number of bins, *B*, is rounded to the nearest integer, called ${\rm Bins}$. This is then used to create a histogram $JD_{{\rm hist}}$ of the elements of $\overline {{\bi JD}} $ with the number of bins calculated Bins. When doing this, the elements of $\overline {{\bi JD}} $ are excluding the diagonal.

This is then normalised to the sum of the histogram values.

### 3.3 JDistEn calculation

Using the normalised $JD_{{\rm hist}}$, called $\rho $, the joint distribution entropy, JDistEn, given by

(8) $${\rm JDistEn} = \left({\displaystyle{{ - 1} \over {\mathop {\log }\nolimits_2 \, {\rm Bins}}}\sum\limits_{t = 1}^{{\rm Bins}} \rho _t \times \mathop {\log }\nolimits_2 \, \rho _t} \right)\eqno\lpar 8\rpar $$

can be found. It is also ensured that $\rho \ne 0$ within this calculation, as this will give an invalid answer due to the logarithm.

The range of JDistEn is $0 \le {\rm JDistEn} \le 1$, where 0 means the two channels are not coupled at all and 1 means they are fully coupled.

The JDistEn results of all channels to each other are stored in a 128×128 matrix corresponding to the coupling between all 128 channels. This is a weighted adjacency matrix with values theoretically ranging between 0 and 1.

### 3.4 Constructing binary adjacency matrices

**Fig. 1:**
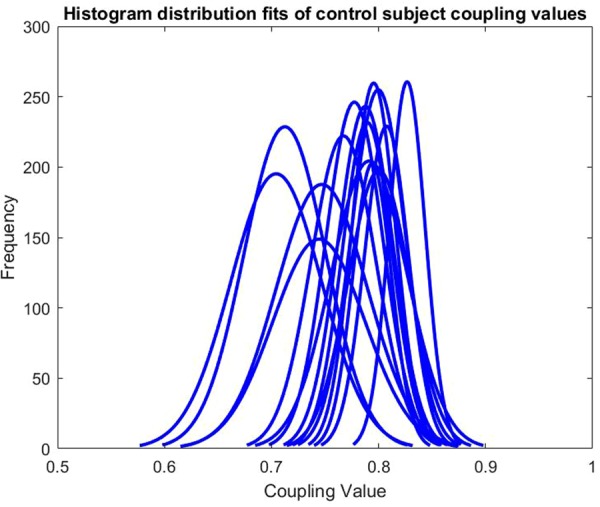
Histogram distribution fits of control subject coupling values

**Fig. 2:**
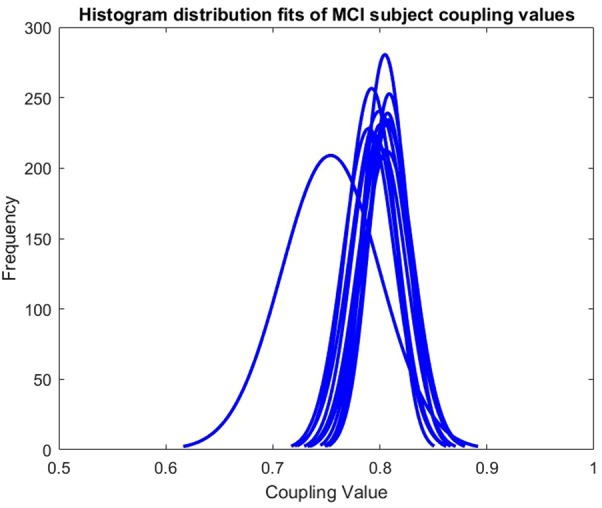
Histogram distribution fits of MCI subject coupling values

For simpler analysis, these adjacency matrices were turned into binary adjacency matrices. The ranges of the coupling values in the different MCI and control subjects’ adjacency matrices varied. This can be seen in Figs. 1 and 2. Therefore, a fixed density method is used to produce a threshold. By this meaning that the proportion being coupled for each subject remains the same. A threshold was set such that the top 10% of each adjacency matrix values were considered coupled, being 1, and the remaining proportion not considered coupled, being 0.

### 3.5 Network parameter analysis

Once networks had been produced for the MCI and control subjects, network parameters can be looked at. The network parameters looked at were the clustering coefficient and characteristic path length, the level of small-world network characteristics, and the eccentricity. Small-world networks can be defined by the clustering coefficient and the average shortest path length (also known as the characteristic path length) [7]. The small-world phenomenon can be quantified into one parameter in the following manner:

(9) $$Q = \displaystyle{{\lpar C_{{\rm actual}}/C_{{\rm random}}\rpar } \over {\lpar L_{{\rm actual}}/L_{{\rm random}}\rpar }}.\eqno\lpar 9\rpar $$

Here, $C_{{\rm actual}}$ and $L_{{\rm actual}}$ are the clustering coefficient and characteristic path length of the network produced using the previously described method.

$C_{{\rm random}}$ and $L_{{\rm random}}$ are the clustering coefficient and characteristic path length of random networks produced. Random networks of the same size were produced with the top 10% of the adjacency matrix being considered coupled in the same manner as the MCI and control subjects’ adjacency matrices were done. Ten networks were created. The clustering coefficient and characteristic path length of the random networks were found. These values were averaged for the ten networks. The averaged values were considered $C_{{\rm random}}$ and $L_{{\rm random}}$. Eccentricity was measured.

### 3.6 Statistical analysis

The validity of the differences seen between the MCI and control subject sets was tested. First, it is checked whether the data are normally distributed through the use of a Jarque–Bera test. If the data are normally distributed, then an unpaired two-sample student's *t*-test can be used. This tests the null hypothesis that the two independent sets come from distributions with the same mean. Simply put, the *t*-test described indicates whether the two sets may be coming from the same distribution. The exact *p*-value is calculated. It is checked whether the null hypothesis is rejected at the 5% significance level. If one or both of the sets are seen to not be normally distributed, a Wilcoxon rank sum test was used instead, as the student's *t*-test work under the assumption that the distribution is normally distributed. This tests a similar null hypothesis which is that the two independent sets come from distributions with equal medians. Similar to the *t*-test, the exact *p*-value is calculated and it is checked whether the null hypothesis is rejected at the 5% significance level.

All results were also checked for outliers using Chauvenet's Criteria and an interquartile range test.

## 4 Results

The histogram distribution fits for the individual subjects can be seen in Figs. 1 and 2. The distribution plots included only the values in the lower triangle of the adjacency matrices as the adjacency matrices are symmetric. The differing ranges of values for different subjects led to the decision of using a fixed density method for thresholding to produce a binary adjacency matrix.

The results can be seen in Table 1. The result from the Jarque–Bera test showed that none of the networks parameters looked at were normally distributed for both the MCI and control set. Therefore, only the rank sum results are shown in Table 1. The results that upheld the 95% certainty threshold were considered to be statistically significant. The statistically significant results are highlighted in bold.

**Table 1:** Comparison of network parameters of beta-filtered MCI and control subjects

	MCI (*n* = 13)		Controls (*n* = 14)		Rank sum
network parameter	mean	SD	mean	SD	p
clustering	0.572475	0.094392	0.645624	0.09575	**0.04938**
characteristic path length	1.940423	0.116031	1.886695	0.071737	0.13886
small-world phenomenon	6.334746	1.080554	7.329532	1.038798	**0.01860**
eccentricity	2.888048	0.468259	2.486873	0.453488	**0.01630**

Statistically significant results are highlighted in bold.

Three differences between the MCI and control group were seen to be statistically significant. First, clustering was seen to be lower in beta-filtered MCI subjects than in beta-filtered control subjects. The small-world phenomenon, which is connected to clustering according to (9), also showed a significant difference between the subject groups. The small-world phenomenon was seen to be less prominent in beta-filtered MCI subjects than in the beta-filtered control subjects. Finally, there was seen to be higher eccentricity in beta-filtered MCI subjects than in the beta-filtered control subjects.

## 5 Discussion

Network parameters in the beta band of MCI and control subjects during a visual short-term memory binding task have been compared. The EEG signals were filtered to retrieve the beta band. A joint distribution entropy method was used to produce adjacency matrices for each subject. The choice of method was particularly important given the short recordings acquired during rapid memory tasks. The non-binary adjacency matrices were transformed into binary adjacency matrices by defining the top 10% in terms of values in each matrix to be coupled and the rest to be non-coupled. The clustering coefficient, characteristic path length, small-world phenomenon, and eccentricity network parameters were then looked at and compared between the MCI subject set and control subject set.

The preliminary results found, which are discussed in this Letter, agree with some of the results seen in other studies. Lower small-world characteristics have been seen in beta-filtered AD patients when compared with controls [9]. Lower small-world characteristics for subjects with AD were found as well as presented in the introduction [10, 13]. The preliminary result of higher eccentricity in beta-filtered MCI subjects is an interesting result that had not previously been analysed much in relation to AD or dementia patients. There may be a correlation between the increased eccentricities that was also seen amongst Parkinson's disease patients [15]. The lower clustering coefficient amongst beta-filtered MCI subjects was supported by some of the other studies. Two studies showed lower clustering coefficient for AD patients in the lower alpha band [13, 14]. So whilst the preliminary result of a lower clustering coefficient in the beta band of MCI subjects sounds promising, it has not been found in the other studies to the best of our knowledge. However, it is crucial to bear in mind that a key characteristic of this Letter is that we analysed data recorded during a visual short-term memory binding task.

There were some limitations with the method used. One potential limitation may be that when producing the adjacency matrices, the number of edges was fixed across subjects, as being 10% of the nodes, excluding connections of nodes to themselves. This fixed edge distribution, means that quantity of particular network parameters may not be comparable across subjects, as this may be merely caused by the fixed density approach. However, it was deemed that this fixed density approach would produce appropriate sparse networks for topological network analysis. However, the selection of a binarising threshold is an active area of research in the analysis of brain functional networks and other alternatives could be considered in the future [23]. Another effect of using the cut-off approach used here is that certain network parameters could not be evaluated.

The results seen give several opportunities for future work. As the difference in eccentricity of beta-filtered MCI and control subjects was statistically significant, it may be interesting to look further into the locations of high or low eccentricity. This way eccentricity could be used to view centrality as well. Instead of averaging eccentricity across nodes, it could be seen which points had lower and higher eccentricity.

It would also be of interest to see if a classifier, which could determine if the subject was an MCI subject, could be formed from the results found in this Letter. Such a classifier would likely include a combination of the network parameters found in this report.

As patients with MCI are more likely to develop dementia, the approach in this Letter could potentially be evaluated in the early detection of dementia which has been highlighted to be of utmost importance in recent years [2]. However, our results are preliminary and limited by the small sample size. Therefore, the algorithms should first be applied to a larger sample to verify the results. If such results were to agree with the ones presented here, then it would be of interest to compare these when the same method is applied to patients with confirmed dementia. This would lead to a better understanding of how dementia develops and the relationship between MCI and dementia.

## 6 Conclusion

Network parameters of beta-filtered MCI subjects can be seen to be different from those of control subjects during a visual short-term memory binding task. The small-world characteristics were seen to be smaller in MCI subjects and the eccentricity was seen to be higher. This Letter contributes to the understanding of EEG activity in MCI during a visual short-term memory binding task.

## 7 Funding and declaration of interests

This research was partially supported by a Knowledge Exchange Fellowship by Alzheimer's Society and Alzheimer Nederland to JE (grant no. 271). Conflict of interests: None declared.
